# Electrochemical Characterization Using Biosensors with the Coagulant *Moringa oleifera* Seed Lectin (cMoL)

**DOI:** 10.3390/bios13060655

**Published:** 2023-06-15

**Authors:** Benny Ferreira de Oliveira, Hallysson Douglas Andrade de Araújo, Eloisa Ferreira Neves, Thiago Henrique Napoleão, Patrícia Maria Guedes Paiva, Katia Cristina Silva de Freitas, Sandra Rodrigues de Souza, Luana Cassandra Breitenbach Barroso Coelho

**Affiliations:** 1 Departamento de Bioquímica, Centro de Biociências, Universidade Federal de Pernambuco (UFPE), Av. Prof. Moraes Rego, 1235, Cidade Universitária, Recife 50670-420, PE, Brazil; bennyfoliveira@gmail.com (B.F.d.O.); hallysson.douglas@ufpe.br (H.D.A.d.A.); thiago.napoleao@ufpe.br (T.H.N.); patricia.paiva@ufpe.br (P.M.G.P.); 2 Departamento de Química, Universidade Federal Rural de Pernambuco (UFRPE), Rua Dom Manuel de Medeiros, s/n, Dois Irmãos, Recife 52171-900, PE, Brazil; eloisaferreira.neves@gmail.com (E.F.N.); katiacsdfreitas@gmail.com (K.C.S.d.F.); souzz.rodrigues@gmail.com (S.R.d.S.)

**Keywords:** lectins, metal–organic frameworks (MOFs), electrochemical systems

## Abstract

Triturated *Moringa oleifera* seeds have components that adsorb recalcitrant indigo carmine dye. Coagulating proteins known as lectins (carbohydrate-binding proteins) have already been purified from the powder of these seeds, in milligram amounts. The coagulant lectin from *M. oleifera* seeds (cMoL) was characterized by potentiometry and scanning electron microscopy (SEM) using MOFs, or metal–organic frameworks, of [Cu_3_(BTC)_2_(H_2_O)_3_]_n_ to immobilize cMoL and construct biosensors. The potentiometric biosensor revealed an increase in the electrochemical potential resulting from the Pt/MOF/cMoL interaction with different concentrations of galactose in the electrolytic medium. The developed aluminum batteries constructed with recycled cans degraded an indigo carmine dye solution; the oxide reduction reactions of the batteries generated Al(OH)_3_, promoting dye electrocoagulation. Biosensors were used to investigate cMoL interactions with a specific galactose concentration and monitored residual dye. SEM revealed the components of the electrode assembly steps. Cyclic voltammetry showed differentiated redox peaks related to dye residue quantification by cMoL. Electrochemical systems were used to evaluate cMoL interactions with galactose ligands and efficiently degraded dye. Biosensors could be used for lectin characterization and monitoring dye residues in environmental effluents of the textile industry.

## 1. Introduction

Lectins are proteins that have binding sites and are able to recognize carbohydrates in a specific and reversible way, thus having biochemical, biomedical and biotechnological applications [1]. They are widely distributed in nature in different tissues of organisms and can adhere to carbohydrates present in the membrane or cell wall, thereby agglutinating cells by recognizing glycoconjugates and complex glycans [2].

Recent research has demonstrated several applications for plant compounds, including as coagulants in water treatment [3,4,5]. *M. oleifera* is a polyvalent tree [6,7,8] with therapeutic properties for alternative medicinal uses [9]. Extracts from its seeds have proven to be more efficient at removing humic acid than a commonly used chemical component [6] and they also have coagulant proteins that can be applied for water treatments and industrial waste removal [10,11], such as the treatment of textile effluents [12].

Experiments with triturated *M. oleifera* seeds, involving spontaneous and exothermic chemical processes to adsorb indigo carmine dye in a liquid medium, have shown excellent results [13].

The environmental impact of the direct discharge of untreated wastewater after the use of dyes is worrying considering the variety and complexity of the chemicals used in the process [14]. Among the many chemical compounds used, dyes have attracted attention due to the high pollution potential they present [15]. In particular, the recalcitrant synthetic dye indigo carmine, widely used in the manufacture of jeans, is difficult to remove from an aqueous medium due to its chemical stability [16]. In Brazil, indigo carmine is broadly applied in the textile industry, especially in the process of customizing jeans, mainly in the northeast region [17].

The discard of textile effluents without treatment into aquatic environments can quickly lead to the depletion of dissolved oxygen, resulting in an imbalance of the ecosystem [18]. The presence of dyes can prevent the penetration of sunlight into the deeper water layers, altering the photosynthetic activity of the medium and resulting in the deterioration of the water quality; the presence of metals and aromatic groups in dyes can induce mutagenic or teratogenic effects, resulting in lethality to aquatic fauna and flora [19,20].

Even with all the problems caused to the environment, the textile industry is of relevance to the economy and various social issues. In Brazil, for, example, it generates 1.34 million formal jobs, and is considered the second largest employer in the manufacturing industry [21] and one of the largest in the world [17].

One of the coagulant molecules of *M*. *oleifera* seeds is a cationic lectin (cMoL), specific for galactose, that has been shown to be resistant to a wide pH range and high temperatures. The partially characterized lectin was revealed to have a molecular profile of 26.5 KDa, an approximate isoelectric point of 11.67, 101 amino acids [22] and the following biological activities: anticoagulant properties [23], insecticidal properties [24], antiparasitic properties [25], cytotoxicity to tumor cells [26] and a clarifying ability of water turbidity similar to aluminum sulfate treatment [22].

Electrochemical methods are sensitive, are financially viable and have a variety of instrument styles and components [27]. These techniques are useful for detecting a diversity of analytes [28,29,30]. Potentiometric and amperometric systems can be used sequentially in the monitoring of industrial waste [31]; they detect, through measurable electrical signals, conformational changes in biomolecules when in contact with a ligand [32].

Electrochemical biosensors are integrated and autonomous devices that use a biochemical receptor to obtain analytical information. These instruments have been designed for several applications [33,34]. Among such applications is the monitoring of water pollutants due to the high sensitivity of biosensors and their application in real time, which are features that have attracted the attention of researchers [35].

Considering that there are different physical, chemical and biological methods for wastewater treatment, it is difficult to choose a single procedure for the efficient removal of dyes from the textile industry [36].

Coagulation corresponds to the first stage of water treatment in the supply networks; it is used to reduce turbidity and coloration, as well as to eliminate pathogens [37]. One of the promising methods for this purpose is electrocoagulation [37,38], which consists of an alternative method to chemical treatment and occurs by destabilizing pollutant particles [39] through redox processes, promoted by an electric current applied in an electrochemical cell. The system is composed of metallic electrodes, which can be iron or aluminum electrolyte solutions [40]. The reaction generates coagulant substances, which can be metallic hydroxides or polyhydroxides, that are responsible for the flocculation of these particles, which float to the surface [41].

With the intention of reducing the cost of electrocoagulation processes for treating contaminated water, the aluminum electrode used in these reactors can be replaced by recycled aluminum beverage cans by building an aluminum battery; the aluminum that constitutes these cans works as an anode [36,38,42].

MOFs, or metal–organic frameworks, comprise two- or three-dimensional structures that form coordination networks with metallic centers and organic ligands [43], which can be presented as nanochannels with adjustable shapes and sizes. Standing out for their qualities, such as the presence of adjustable pores and their large surface area, they have several applications, such as gas separation, emission purification and drug encapsulation [44]. Biosensors that use nanomaterials are paving the way for the development of devices with a better performance for monitoring environmental parameters, such as water quality control [45].

In this work, cMoL was characterized by a potentiometric biosensor and scanning electron microscopy (SEM). In addition, an amperometric biosensor was designed using cMoL immobilized in an MOF of [Cu_3_(BTC)_2_(H_2_O)_3_]_n_, with the objective of monitoring residual indigo carmine dye by simulating a textile effluent degraded by electrocoagulation.

## 2. Materials and Methods

### 2.1. Lectin Isolation

An extract obtained from *M. oleifera* seeds in powder and a saline solution was added to ammonium sulfate at 60% (*w*/*v*) saturation according to Green and Hughes [46]. The precipitated fraction (F 0–60%) was collected, resuspended and dialyzed with distilled water (4 h, with two liquid changes); it was then added to a column (10 × 1.0 cm) containing guar gel [47] and equilibrated with 0.15 M NaCl. The chromatographic conditions used were the same as those reported by Santos et al. [22] to purify cMoL. A flow rate of 20 mL/h was maintained and, after a washing step with the equilibration solution, the adsorbed proteins were eluted with 1.0 M NaCl. Fractions of 2.0 mL were collected and evaluated for their absorbance at 280 nm and their hemagglutinating activity (HA). The protein concentration was estimated using the method of Lowry et al. [48] and an HA assay was used to evaluate the carbohydrate binding capacity of the lectin, according to Paiva and Coelho [49].

### 2.2. Electrochemical Synthesis of MOF [Cu_3_(BTC)_2_(H_2_O)_3_]_n_

The MOF [Cu_3_(BTC)_2_(H_2_O)_3_]_n_ was a porous and crystalline polymer composed of a metallic center, copper dimers (two copper II ions) and a paddlewheel unit formed by four carboxylate anions of the BTC ligands. It was synthesized electrochemically according to the method developed by Silva et al. [50] using a solution of 1,3,5-benzenetricarboxylic acid, sodium nitrate and dimethylformamide in Millipore^®^ water in a 1:1 ratio. The synthesis lasted about 17 min and a blue-colored solid was obtained.

### 2.3. Preparation of the Working Electrode

The working electrode consisted of a platinum plate with an area of 0.5 cm^2^. Before mounting, it was subjected to chemical washing with immersion in a solution of nitric acid (HNO_3_) P.A. for 2 min, and was then washed with distilled water and dried at room temperature.

In the immobilization process of lectin in the MOF [Cu_3_(BTC)_2_(H_2_O)_3_]_n_, 0.0060 g of this polymer was weighed and 20 μL of cMoL 3 mg/L was added at a temperature of 4 °C for 24 h. To fix the MOF/cMoL on the platinum electrode, a paste composed of 0.0650 g of powdered carbon and 6 drops of mineral oil was used to build the modified working electrode, Pt/MOF/cMoL.

### 2.4. Potentiometric Measurements

After assembling the working electrode under the conditions mentioned above and in Section 2.3, measurements of the electrochemical potentials of the following steps were taken:

(1) Platinum electrode with MOF (Pt/MOF);
(2) Platinum electrode with Pt/MOF/cMoL with 0.15 M NaCl in the electrolytic medium;
(3) Pt/MOF/cMoL with 0.15 M NaCl and different concentrations of galactose: 10, 15 and 20 mM.

The working electrode, together with the silver/silver chloride (Ag/AgCl) reference electrode and the saline solution in the electrolytic medium, constituted the electrochemical cell; this system was connected to a digital voltmeter with a scale of 0–1000 V.

For each treatment, three samples were used and the experiments were repeated five times.

### 2.5. Scanning Electron Microscopy

For the characterization of MOF crystals, images were obtained from samples of MOF crystals with immobilized cMOL, MOF crystals with immobilized cMOL interacting with 10 mM galactose and MOF crystals interacting with 10 mM galactose, using a scanning electron microscope (SEM) and other equipment (TESCAN, Model: VEGA3) from the CENAPESQ/UFRPE.

### 2.6. Electrocoagulation

For the construction of an aluminum battery, an indigo carmine solution was used at a concentration of 0.1 g/L^−1^. In each recycled can with copper wires (12.5 cm long and 2.5 mm thick), 0.15 M sodium chloride was added to the dye solution.

The components of the electrochemical cell were iron electrodes (sacrifice) and an electrolyte solution, and the cell contained the same solution as the aluminum battery.

**Figure 1:**
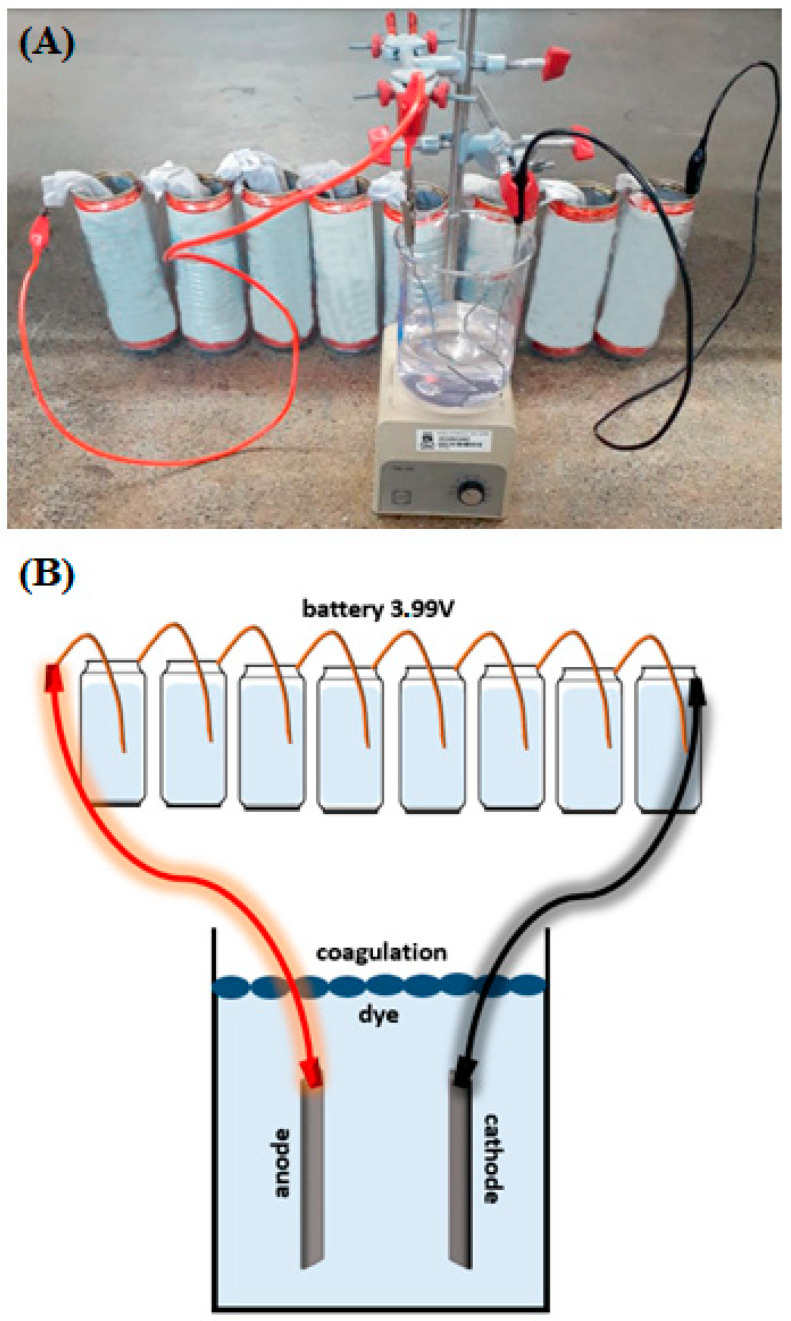
Electrochemical system showing the mechanism of electrocoagulation, resulting from reactions occurring at the anode and cathode (**A**); representative scheme of the aluminum battery (**B**).

The system was connected to an external energy source (aluminum battery) composed of batteries, whose electrodes were aluminum containers (anode) and copper wires (cathode); these batteries were connected in series, for which the copper was inserted inside each container by connecting it to the next container. The potential was measured by coupling the positive terminal of the multimeter to the first copper wire and the negative terminal to the last container, and was equivalent to the sum of the potential of each battery (Figure 1). The treatment of the solution with dye in these recycled aluminum cans lasted for 1 h.

### 2.7. Cyclic Voltammetry

The electrochemical characterization was carried out using cyclic voltammetry in the potentiostat/galvanostat of Autolab Eletrochemical Instruments, located in the Laboratory of Chemical Analysis and Sensors–LAQIS/UFRPE. The measurements of cyclic voltammetry used the Ag/AgCl reference electrode, a platinum wire as a counter electrode and the modified working electrode Pt/MOF/cMoL inserted into samples containing indigo carmine after treatment with recycled aluminum cans. All measurements were derived from five replicates.

## 3. Results and Discussion

Potentiometric measurements were performed using the working electrode and the reference electrode, both immersed in a 0.15 mol/L NaCl solution and coupled to a potentiometer.

**Figure 2:**
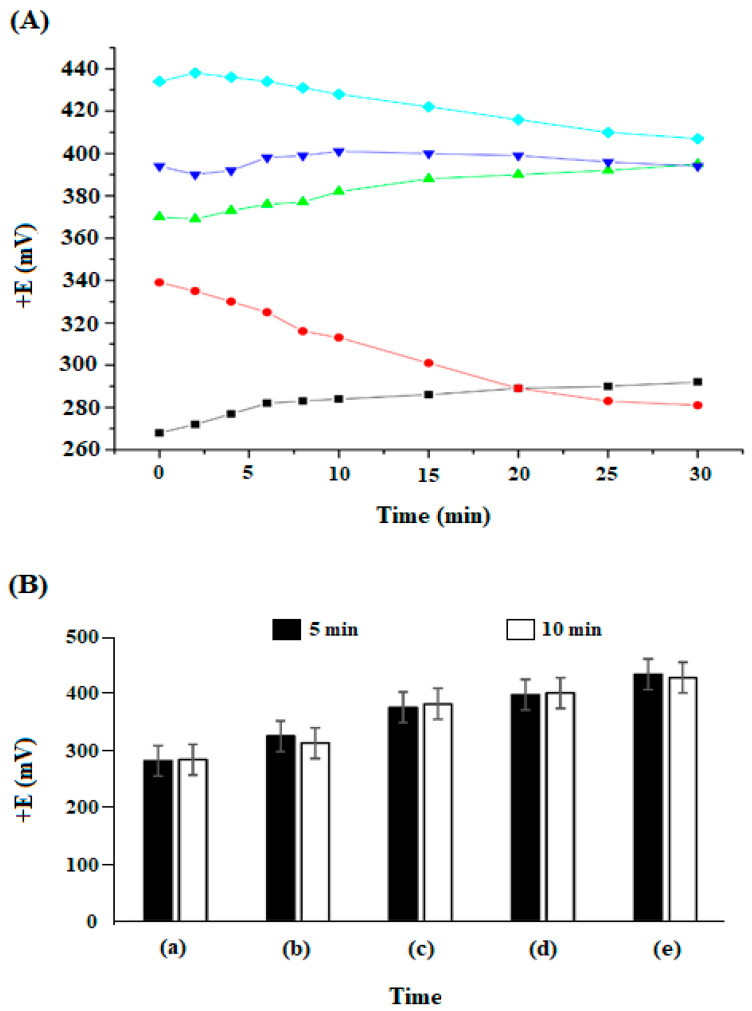
(**A**) Electrochemical potential of platinum electrode coated with MOF (Pt/MOF) (-■-); platinum electrode coated with MOF and immobilized cMoL (Pt/MOF/cMoL) (-•-); and platinum electrode coated with MOF and immobilized cMoL (Pt/MOF/cMoL) and interacting with different concentrations of galactose: 10 mM (-▲-), 15 mM (-▼-) and 20 mM (-♦-). (**B**) Application of bar errors (5%) in the results obtained at intervals of 5 and 10 min: electrochemical potentials of platinum electrode coated with MOF (Pt/MOF) (a); platinum electrode coated with MOF and immobilized cMoL (Pt/MOF/cMoL) (b); and platinum electrode coated with MOF and immobilized cMoL (Pt/MOF/cMoL) and interacting with different concentrations of galactose: 10 mM (c), 15 mM (d) and 20 mM (e).

The electrochemical potential was verified in all phases of this analytical system (Figure 2A,B). Higher electrochemical potentials resulted from the interactions between cMoL immobilized on the MOF-coated platinum electrode (Pt/MOF/cMoL) and the different concentrations of galactose. The efficiency of cMoL immobilization in the metal–organic structure [Cu_3_(BTC)_2_(H_2_O)_3_]_n_ contributed to the stability of the biosensor, thus facilitating the electrochemical potentials and showing that the use of an electrochemical system with a modified working electrode can be applied to study the interaction of cMoL with galactose.

In the electrochemistry of biological molecules, a biosensor is considered efficient when the immobilized biological element does not undergo denaturation, thereby maintaining its activity [44,51,52]. According to Martínez-Pérez-Cejuela et al. [53], the combination of the MOF with luciferase contributed to the conformational stability of this enzyme, facilitating substrate access in a paper bioluminescence biosensor to monitor ATP; in this way, it contributed to the constancy of the data obtained by this device. This biocompatibility was also observed in electrochemical biosensors using polymers such as MOFs and covalent organic frameworks (COFs) in assays with enzymes and antibodies [32].

The electrochemical potential was verified in all phases of this analytical system at different concentrations of galactose, with the results observed over a time period of 5 and 10 min (Figure 2A); the mathematical model of bar errors (5%) was applied for these time parameters (Figure 2B). A significant increase was revealed in the electrochemical potential for this time range in relation to the addition of carbohydrates in the electrolytic medium.

The characterization of *Craylia mollis* seed lectin (Cramoll), immobilized on a gold electrode coated with the crystalline polymer MOF [Cu_3_(BTC)_2_(H_2_O)_2_]_n_ with different concentrations of glucose in the electrolytic medium, induced an increase in the electrochemical potential, which was also detected between 5 and 10 min [54].

However, no variation was observed for each specific carbohydrate concentration between the respective analysis times (5 and 10 min), due to the stabilization of the potential generated by the platinum electrode coated with MOF (Pt/MOF/cMoL) (working electrode) in relation to the reference. A significant elevation in these electrochemical potentials was verified by the increase in the concentration of galactose in the electrolyte medium, revealing that this rise in the magnitude of the electrical signal produced in the system was the result of intrinsic conformational changes that occurred on the surface of the immobilized lectin, without altering the native structure of cMoL. The biocompatibility exhibited by MOF for the manufacture of electrochemical biosensors, with a good performance, was generated by the combination of MOF/cMOL.

The biological performance of lectin correlating structural modifications and surface charge distribution was elucidated by potentiometry [54]. This redox electroactivity can reveal data characteristics of the equilibrium state through electrochemical methods; the application interface with electrical charges, adsorbed on the surface of the electrode, can be evaluated in a simplified way [32].

The redox-active nature of MOFs favors their application in electrochemical biosensors [43]. This property has an effect on the charge distribution on the surfaces of biomolecules in electrochemical systems that is relevant to understanding their interaction mechanisms and biological properties [55].

This elevation of the electrical signal generated through the electrodes of a biosensor by increasing the concentration of the analyte in an electrochemical cell has been confirmed by Carvalho et al. [54], Mohankumar et al. [33] and Selzer et al. [55].

This electrochemical model with the lectin immobilized in the MOF for the construction of a biosensor has also been verified by Carvalho et al. [54] for evaluating the results of the electrochemical potentials related to the interaction of Cramoll immobilized in an MOF with different concentrations of glucose. The combination of lectins with semiconductors, such as metallic polymers, increases their recognition capacity in biosensors [56].

The immobilization of lectins for sugar recognition offers further advantages among biospecific protein/carbohydrate systems, as they do not undergo structural changes after binding and they can also provide independent carbohydrate-binding domains [57,58].

**Figure 3:**
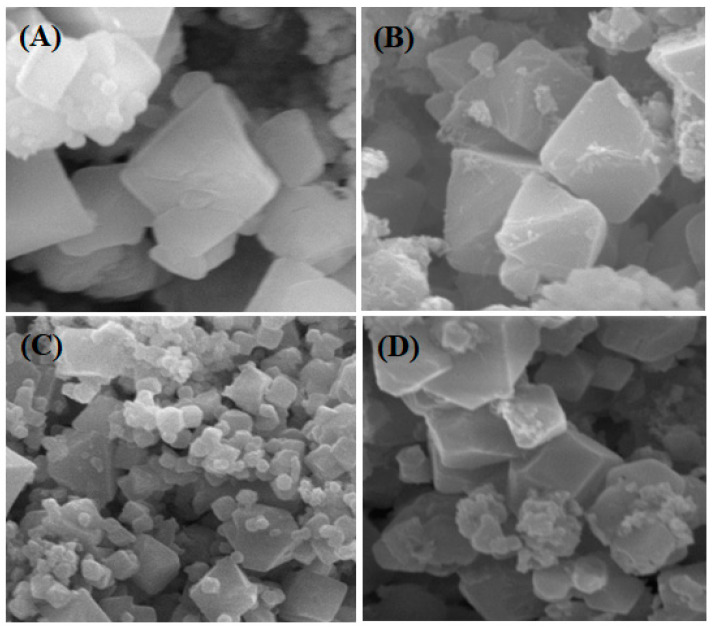
[Cu_3_(BTC)_2_(H_2_O)_3_]_n_ MOF crystals (**A**), [Cu_3_(BTC)_2_(H_2_O)_3_]_n_ MOF crystals with immobilized cMoL (**B**), [Cu_3_(BTC)_2_(H_2_O)_3_]_n_ MOF crystals with immobilized cMoL interacting with 10 mM galactose (**C**), and [Cu_3_(BTC)_2_(H_2_O)_3_]_n_ MOF crystals with 10 mM galactose (**D**).

In addition to the electrochemical biosensor, scanning electron microscopy (SEM) was used to characterize the MOF and investigate its interactions with cMoL and galactose (Figure 3).

The geometry of the MOF Cu-BTC crystals is represented by a smooth octahedral structure, approximately 2 μm in size. Figure 3B shows galactose adhered to the MOF surface; in Figure 3C, a globular structure of cMoL adhered to the MOF surface indicates the efficiency of immobilization on the surface of this metal–organic framework and the complete MOF/cMoL/galactose system is represented in Figure 3D. This interaction model was confirmed by the evolution of electrochemical potentials, as shown in Figure 2.

SEM is a perfect technological tool that helps with the characterization of biomolecules immobilized on MOFs due to the production of high-resolution images, which are capable of showing the target surface being analyzed [43,54,59,60].

After cMoL characterization by a potentiometric biosensor and the analysis of the images generated by SEM, a system of aluminum batteries was developed for the degradation of indigo carmine dye in an electrolytic medium through the process of electrocoagulation.

The success of electrocoagulation is already known for treating wastewater, such as recycling water contaminated by domestic sewage [38]. The advantages of this method over others that use an electric current to coagulate suspended solids in contaminated water are a reduction in energy consumption and a reduction in operating costs [36,38,42].

The treatment of dye residues in aluminum batteries is shown by the electrocoagulation mechanism (Figure 1). At the battery’s cathode, oxygen dissolved in water undergoes reduction on the copper surface, forming hydroxyl. At the anode, aluminum undergoes oxidation, releasing Al^3+^, which reacts with the hydroxyl to form Al(OH)_3(s)_, a white precipitate responsible for the coagulation of the dye contained in the solution, according to the following equations:

Anode: Al_(s)_ → Al^3+^_(aq)_ + 3e^−^, E^0^ = −1.66 V (1)

Cathode: O_2_ + 2H_2_O + 4e^−^ → 4OH^−^, E^0^ = 0.82 V (2)

The potential generated by the battery was equivalent to 3.99 V and allowed electrolysis to treat new dye samples in the system formed by the beaker and iron electrodes (Figure 1). The container was the anode in the battery because it underwent oxidation. The electrons formed in this oxidation traveled through the external wire to the iron electrode, which promoted the reduction of water, forming hydroxide (OH^−^) and hydrogen (H_2_), while in the electrode of the iron connected to the copper wire, oxidation of the iron occurred, forming Fe^2+^, which reacted with the hydroxide to produce Fe(OH)_2_, a gelatinous precipitate capable of coagulating the dye contained in the solution.

Fe_(s)_ → Fe^2+^_(aq)_ + 2e^−^ (3)

2H_2_O + 2e^−^ → H_2_ + 2OH^−^ (4)

The pH of the samples with dye was measured to verify the formation of hydroxides in the solution (Table 1).

The pH values of the samples were measured before and after the electrochemical treatment with iron and aluminum electrodes.

There was an increase in the pH of the samples after treatment, suggesting that this increase in pH occurred due to the evolution of hydrogen in the cathode [61]. Our results corroborate the studies presented by Lach et al. [62], who observed an increase in the pH value for the textile effluent in the electrocoagulation process with aluminum electrodes. The techniques commonly applied in the treatment of effluents have a high cost of chemical components, thus stimulating the search for new methods of water treatment [63]. Electrocoagulation methods are more advantageous than other treatments, since they use simple, easy-to-handle instrumentation, and the residual sludge formed can be retained more easily [64]; therefore, this technique is widely used to remove color and decontaminate effluents [65]. Despite this, the method has some limitations, such as electricity consumption, so new improvement alternatives have been investigated [66]. Therefore, the system composed of recycled aluminum cans generating energy to activate the degradation reaction of the dye represents a development of economic and environmental importance.

Based on the results obtained (Figure 2) and carefully substantiated by the literature, it appears that the immobilization of cMoL in an MOF of [Cu_3_(BTC)_2_(H_2_O)_3_]_n_ was efficient and facilitated the obtaining of electrochemical potentials, showing that the electrochemical system used with a modified working electrode can be applied to study the interaction of this lectin with indigo carmine dye.

In order to build a biosensor to detect dye residues in samples after electrocoagulation with aluminum batteries, cMoL was used to aggregate MOF crystals on a platinum electrode surface, providing stability for the use of this device. This aggregate effect was also verified with Cramoll lectin on the surface of a gold electrode containing an MOF (54).

**Figure 4:**
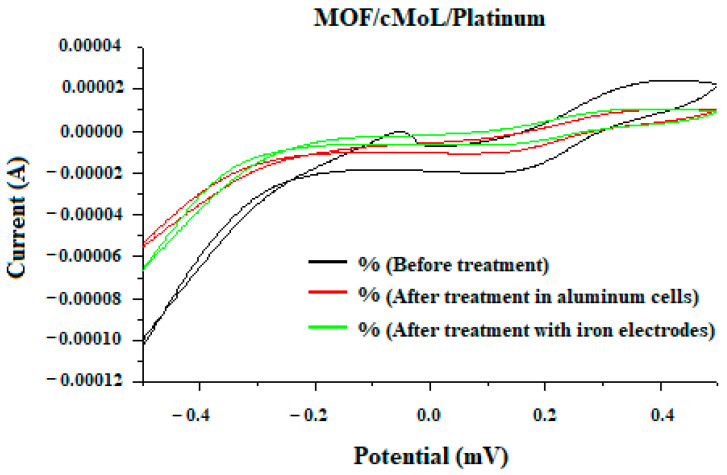
Cyclic voltammogram with samples before treatment, after treatment in the aluminum cell and after treatment by electrolysis with iron electrodes.

To identify the occurrence of interactions between cMoL and the indigo carmine dye, cyclic voltammetry was performed (Figure 4). According to Mohankumar et al. [33], the oxidation and reduction peaks shown in cyclic voltammograms result from the current variation generated on the surface of the electrodes in an electrochemical biosensor.

Electroanalytical methods such as cyclic voltammograms using electrodes modified with nanocomposites are increasingly being required to identify the redox properties of biomolecules [67], such as MOFs, due to their biocompatibility for glucose and H_2_O_2_ detection [43].

In this work, through a voltammogram, the treatment efficiency was observed due to the difference in the redox peaks, for which there was a decrease in the current peaks in the treated samples, indicating that cMoL was able to monitor the residues before and after the treatment. No significant difference was verified between the samples treated in aluminum batteries or after electrolysis. In a previous study, cMoL was purified and showed a coagulant capacity for removing impurities in turbid water [22].

The peaks that stood out in the cyclic voltammograms were due to the redox process of the MOF/cMoL interaction with the dye in the untreated samples.

This is the first time that an MOF/cMoL/platinum amperometric biosensor has been utilized to detect dye residues after treatment with aluminum batteries and electrolysis, representing a new innovation to control water residues. Electrocoagulation, in addition to its recognized ability to purify contaminated water from industrial effluents, avoids the risk of secondary pollution of the treated material, as it does not require additional chemical products [68].

Thus, this operating system, using electrocoagulation with aluminum batteries and an MOF/cMoL amperometric biosensor with the function of monitoring the purification process of the electrolytic medium contaminated by indigo carmine, appears to be efficient for treating industrial textile effluents.

Electrochemical biosensors are currently considered a high-technology system for detecting target analytes due to their ease of handling, sensitivity, selectivity, speed and the reproducibility of their responses [32]; due to this specificity and sensitivity, these biosensors have excellent applicability for determining contaminants that cause water pollution [45].

The electrocoagulation method was efficient at removing the dye, indicating the possibility of using systems containing reactors with aluminum and copper electrodes for the treatment of effluents from the textile industry. A biosensor using cMoL can be applied for the recognition of dyes in textile industry effluents.

## 4. Conclusions

The coagulant lectin from *M. oleifera* seeds (cMoL) was characterized by the electrochemical method. The potentiometric data obtained demonstrated the interaction of cMoL with different concentrations of galactose, as a consequence of the conformational changes that occurred on the lectin surface. The electrocoagulation method was efficient at removing the indigo carmine dye and constitutes a conventional system. Furthermore, the biosensor using MOF/cMoL was able to monitor dye residues in samples treated with iron and aluminum electrodes.

The treatment on an industrial scale of effluents contaminated with dyes requires a fast and efficient process, due to the negative effect of these substances on the environment. Aluminum battery reactors for the treatment of wastewater contaminated with synthetic indigo carmine dye are promising for use in the textile industry, as they represent a low-energy-cost technique, with easy handling, using recycled aluminum cans. An amperometric MOF/cMoL biosensor will allow quick and easy dye detection.

## Figures and Tables

**Table 1:** Measurement of the pH values of the samples.

Samples with Dye	pH
Sample without treatment	5.97
Sample after aluminum electrode treatment	6.24
Sample after iron electrode treatment	7.01

## Data Availability

The data presented in this study are available on request from the corresponding author.
